# Dynamic Mechanical Properties and Modified Johnson-Cook Model Considering Recrystallization Softening for Nickel-Based Powder Metallurgy Superalloys

**DOI:** 10.3390/ma17030670

**Published:** 2024-01-30

**Authors:** Chen Ling, Xiaoping Ren, Xuepeng Wang, Yinghao Li, Zhanqiang Liu, Bing Wang, Jinfu Zhao

**Affiliations:** 1 School of Mechanical Engineering, Shandong University, Jinan 250061, China; sdulingchen@mail.sdu.edu.cn (C.L.); sduwangxuepeng@mail.sdu.edu.cn (X.W.); 202334403@mail.sdu.edu.cn (Y.L.); melius@sdu.edu.cn (Z.L.); sduwangbing@sdu.edu.cn (B.W.); sduzhaojinfu@sdu.edu.cn (J.Z.); 2 Key National Demonstration Center for Experimental Mechanical Engineering Education, Key Laboratory of High Efficiency and Clean Mechanical Manufacture of MQE, Jinan 250014, China

**Keywords:** dynamic mechanical properties, constitutive model, dynamic recrystallization, powder metallurgy superalloy

## Abstract

The material undergoes high temperature and high strain rate deformation process during the cutting process, which may induce the dynamic recrystallization behavior and result in the evolution of dynamic mechanical properties of the material to be machined. In this paper, the modified Johnson-Cook (J-C) model for nickel-based powder metallurgy superalloy considering dynamic recrystallization behavior in high strain rate and temperature is proposed. The dynamic mechanical properties of the material under different strain rates and temperature conditions are obtained by quasi-static compression test and split Hopkinson pressure bar (SHPB) test. The coefficients of the modified J-C model are obtained by the linear regression method. The modified model is verified by comparison with experimental and model prediction results. The results show that the modified J-C model proposed in this paper can accurately describe the mechanical properties of nickel-based powder metallurgy superalloys at high temperatures and high strain rates. This provides help for studying the cutting mechanism and finite element simulation of nickel-based powder metallurgy superalloy.

## 1. Introduction

Compared with cast and wrought superalloy, the powder metallurgy (PM) superalloy has been widely used in advanced aero-engine turbine disks [1,2] due to its homogeneous microstructure, fine grains, higher yield strength and no mace segregation [3,4], etc. Although PM is a near-forming process, machining is still a necessary process to meet the final accuracy requirements. However, due to low thermal conductivity and low elastic modulus, PM superalloys are deemed very difficult to machining materials [5,6]. Among them, FGH96 is a typical γ’-phase strengthened nickel-based powder superalloy, which often works at a high temperature of about 750 °C. Since FGH96 has a series of characteristics of PM superalloys, there are some challenges in studying the mechanical properties of FGH96 during the cutting process.

The external power is converted into deformation energy in the cutting process. At the same time, the heat is not easy to release, causing the tool and workpiece material temperature rise, resulting in material softening [7]. On the other hand, with the increase in strain, the material work hardening occurs. The interaction between the two makes the material strength not only a function of strain, but also a function of strain rate and deformation temperature, which is called the constitutive relation equation [8,9]. Establishing the accurate constitutive relationship of the workpiece material in the cutting deformation zone is the key to studying the cutting deformation mechanism.

The constitutive equation for the cutting process is mainly divided into two categories, namely, phenomenological models and physical models [10]. Compared with physical models, phenomenological models have become widely used due to their simple form and wide applicability [11]. As the most widely used phenomenological model, the Johnson-Cook (J-C) model [12] is given in the form of a three-part product, representing strain hardening, strain rate hardening, and thermal softening, respectively. However, the above three items of the J-C model are considered to be independent of each other, and the effects of the microstructure of the material itself and the adiabatic temperature rise on the flow stress are not considered [13,14,15].

The occurrence of dynamic recrystallization in the cutting process has been confirmed by researchers [16,17]. Dynamic recrystallization not only refines the grain size of the workpiece material, but also produces a softening phenomenon that cannot be ignored in the flow stress of the material during the deformation process.

A constitutive equation that can accurately describe the microscopic deformation process is still to be realized. Calamaz M et al. [18] established a JC-TANH modified model to describe the phenomenon of soft flow caused by dynamic recrystallization of cutting titanium alloy materials and applied it to the finite element software. This model was verified by Ulutan [18] in finite element simulation. Other similar corrections to the J-C constitutive equation can be found in the literature [19,20].

However, the above modified models only use the temperature (*T_m_*/2) condition as the critical condition for recrystallization. According to the modified J-C model of Denguir et al. [21], the recrystallization strain threshold was proposed. This critical condition considers the combined effects of temperature and strain rate.

The cutting process is a strong coupling process of transient high strain, high strain rate, and temperature. The occurrence of recrystallization is very important to the dynamic mechanical properties of PM superalloy. However, the current research on the modified constitutive equation considering recrystallization for difficult-to-machine materials focuses on titanium alloys. Research on the dynamic mechanical properties of PM superalloy at high temperatures and high strain rates, especially the influence of dynamic recrystallization, is still very scarce.

This paper analyzed the dynamic mechanical properties of powder metallurgy superalloy by quasi-static test and SHPB test. A modified constitutive model considering the dynamic recrystallization behavior of the cutting process is established. The parameters of the modified model are obtained by linear fitting of the experimental data. Finally, it is verified that the model has good accuracy.

## 2. Experimental

### 2.1. Specimen Preparation

The FGH96 superalloy used in this study is prepared by hot isostatic pressing (HIP) and its composition is shown in Table 1. The experiment method consists of two parts, which are quasi-static state compression tests and SHPB tests.

The sample sizes for quasi-static state compression tests and SHPB tests are *φ*3 mm × 3 mm and *φ*2 mm × 2 mm, respectively. By grinding the end face and outer surface of the sample obtained by wire cutting, the sample has higher dimensional accuracy and lower surface roughness (*R_a_* ≤ 0.8 mm). In addition, it is necessary to ensure that the parallelism of the two ends is less than 0.01 mm, to reduce the experimental error caused by the sample.

### 2.2. Quasi-Static State Compression Tests

**Figure 1:**
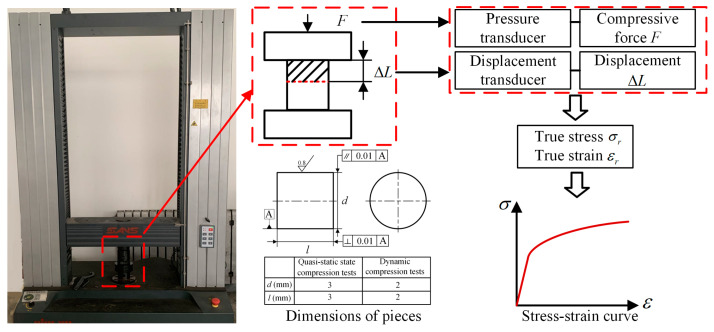
Schematic diagram of quasi-static compression test device.

The quasi-static compression test of the FGH96 superalloy is carried out in the electric universal testing machine. Figure 1 is the test device and schematic diagram. All the experiments are carried out at room temperature. All pieces were subjected to unidirectional compression.

The strain rate of the quasi-static compression test is set to 0.001 s^−1^ and the compression rate can be obtained as 0.18 mm/min according to Equation (1). The compressive force *F* and displacement ΔL are obtained by the pressure transducer and displacement transducer, and the true stress *σ_r_* and true strain *ε*_r_ of the material in the process of compression deformation can be obtained according to the size of the sample. To ensure the reliability of the data, three repeated tests were carried out for each specimen, and the average value was obtained.

(1) $$\dot{\varepsilon }=\frac{d\varepsilon }{dt}=\frac{v}{h}$$

### 2.3. SHPB Tests

**Figure 2:**
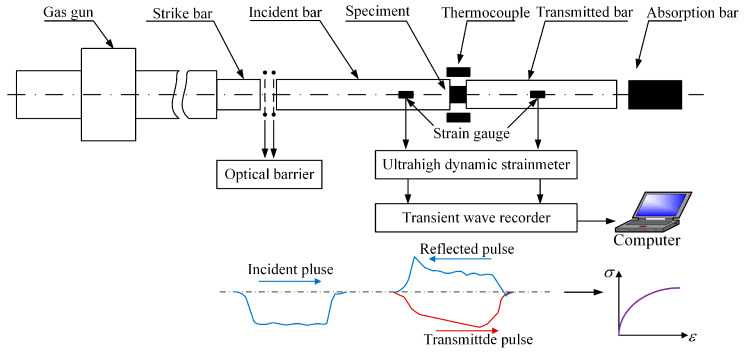
Schematic diagram of SHPB equipment system.

The SHPB equipment system is schematically illustrated in Figure 2. To obtain a higher strain rate, the diameter of the incident bar and the transmitted bar is reduced to 5 mm. By adding different air pressures to the gas bar to change the speed of the strike bar, different strain rate conditions are achieved. Considering the softening effect of high temperature on the material, the same strain rate can be achieved by appropriately reducing the air pressure. Different loading temperatures are controlled by the thermocouple. To make the sample fully heated, it is necessary to carry out heat preservation treatment. In addition, the error caused by the friction between the specimen and the compression bar is reduced by uniformly applying a high-temperature-resistant lubricant on the contact surface between the sample and the rod.

The wave velocity of the incident rod is recorded by the velocimeter, and the incident, transmitted, and reflected pulses are recorded by the strain gauge. According to the one-dimensional stress wave theory, the engineering strain *ε_eng_*, engineering stress *σ_eng_*, and strain rate ${\dot{\varepsilon }}_{eng}$ sample can be calculated by Equations (2)–(4).

(2) $$\varepsilon_{eng}\left(t\right)=-\frac{2c_{0}}{l_{0}}\int_{0}^{t}\varepsilon_{r}\left(t\right)dt$$

(3) $$\sigma_{eng}=\frac{AE\varepsilon_{t}}{A_{0}}$$

(4) $${\dot{\varepsilon }}_{eng}=-\frac{2c_{0}\varepsilon_{r}}{l_{0}}$$

where *c*_0_ is the wave speed in the incident bar, *E* and *A* are Young’s modulus and the cross-sectional area of the bars. *A*_0_ and *l*_0_ are the cross-sectional area and the length of the cylindrical specimen. *ε_r_* and *ε_t_* are the reflected and transmitted strains, respectively, when the reflected and transmitted waves propagate independently.

Under the condition that the material is incompressible, the true strain *ε_T_*, and true stress *σ_T_* of the specimen can be obtained, by Equations (5) and (6).

(5) $$\varepsilon_{T}\left(t\right)=-\ln\left(1-\varepsilon_{eng}\right)$$

(6) $$\sigma_{T}=\sigma_{eng}\left(1-\varepsilon_{eng}\right)$$

Considering the strain rate and temperature in the actual cutting process and the applicable conditions of the experimental device, the experimental conditions of SHPB tests are shown in Table 2. To ensure the accuracy of the experimental data, three repeated experiments were carried out under the same conditions, and the holding time and air pressure of each group of repeated experiments were controlled to be consistent.

## 3. Modified Constitutive Model

The modified model proposed in this paper considers the coupling effect of strain, strain rate, and temperature and the influence of microstructure transformation based on the J-C model, as shown in Equation (7).

(7) $$\sigma =(A+B\varepsilon^{n})(1+C\ln\frac{\dot{\varepsilon }}{{\dot{\varepsilon }}_{0}})\left[1-{\left(\frac{T-T_{0}}{T_{m}-T_{0}}\right)}^{m}\right]H(\varepsilon ,\dot{\varepsilon },T)$$

where *A* represents the yield stress, *B* and *n* represent the material strain hardening coefficients, *ε* represents the true strain, *C* represents the viscosity coefficient, represents the strain rate, *ε*_0_ represents the reference strain rate, *T* represents the temperature, *T_m_* represents the material melting temperature, *T*_0_ represents the room temperature, and *m* represents the thermal softening coefficient. The effect of dynamic recrystallization on flow stress is characterized by adding a correction item $H(\varepsilon ,\dot{\varepsilon },T)$ to the J-C model. The mathematical expressions of $H(\varepsilon ,\dot{\varepsilon },T)$ are shown in Equations (8)–(10). Considering that the recrystallization threshold is affected by both temperature and strain rate, it is expressed by Equation (11).

(8) $$H(\varepsilon ,\dot{\varepsilon },T)=\frac{1}{1-h(\varepsilon ,\dot{\varepsilon })\cdot u(\dot{\varepsilon },T)}$$

(9) $$u(\dot{\varepsilon },T)=\left\{\begin{array}{l} 0,\varepsilon <\varepsilon_{r}\\ 1,\varepsilon \geq \varepsilon_{r} \end{array}\right.$$

(10) $$h(\varepsilon ,\dot{\varepsilon })=\frac{h_{0}}{\varepsilon }+h_{1}-(\frac{h_{0}}{\varepsilon }+h_{2})\ln\left(\frac{\dot{\varepsilon }}{{\dot{\varepsilon }}_{0}}\right)$$

(11) $$\varepsilon_{r}=r_{0}+r_{1}T^{r_{2}}+r_{3}{\dot{\varepsilon }}^{r_{4}}+r_{5}T^{r_{2}}{\dot{\varepsilon }}^{r_{4}}$$

where *h_i_* (*i* = 0, 1, 2) and *r_i_* (*i* =0, 1…5) are material constants, *ε_r_* is the critical strain for the occurrence of dynamic recrystallization, which is expressed by Equation (11).

## 4. Results and Discussion

### 4.1. Dynamic Mechanical Properties

#### 4.1.1. Characteristics of the Stress–Strain Curve

**Figure 3:**
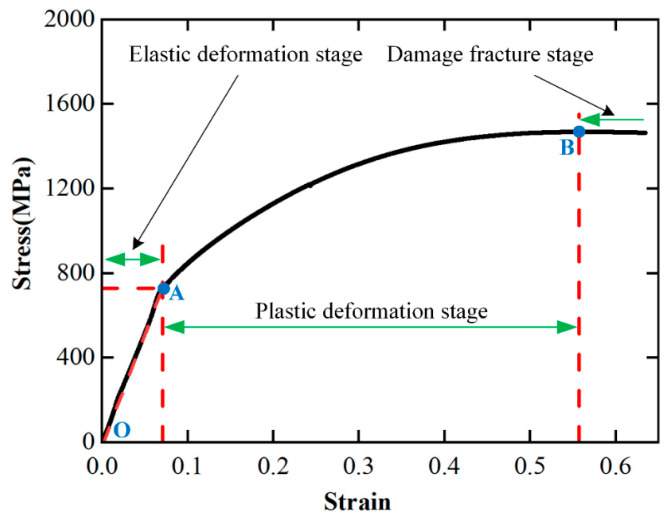
Stress–strain curve of FGH96 superalloy in the quasi-static compression test.

The stress–strain curve of the FGH96 superalloy obtained by the quasi-static compression test is shown in Figure 3. According to Figure 3, in the first stage of the compression process (*OA*), the material undergoes elastic deformation with the stress increases linearly with the increase in strain. When the strain reaches point *A*, the growth rate of stress slows down with the increase in strain, indicating that the material enters the plastic deformation stage (*AB*), showing a clear work hardening phenomenon. When the strain exceeds the strain at point *B*, the material enters the damage deformation stage which is marked by the decrease in stress with the increase in strain.

According to the above analysis, there is no clear yield phenomenon in the quasi-static compression process of the FGH96 superalloy. The yield stress of bulk samples was measured by 0.2% strain shift method, namely, the stress value when 0.2% plastic deformation occurs is taken as its yield strength (point *A* in Figure 3). The yield strength of the FGH96 superalloy is *σ*_0.2_ = 773 MPa.

**Figure 4:**
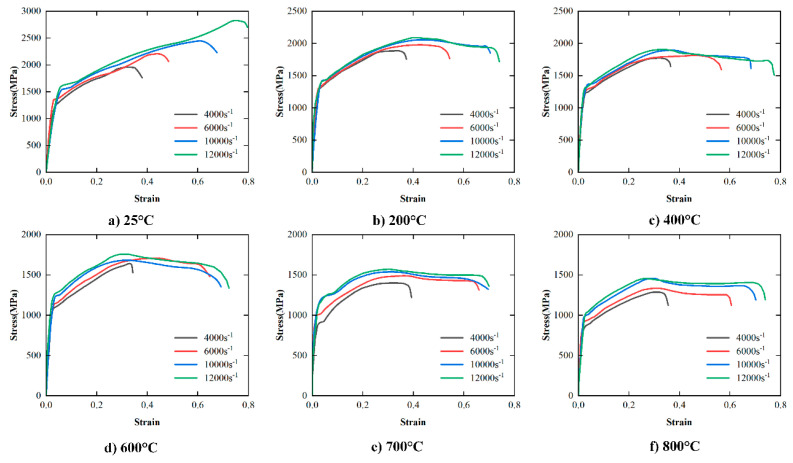
Stress–strain curves of FGH96 superalloy over the wide range of temperature in the SHPB tests.

The effect of strain rate on the stress–strain curve of FGH96 superalloy obtained by SHPB tests at different temperatures (25 °C, 200 °C, 400 °C, 600 °C, 700 °C, 800 °C) is shown in Figure 4. According to Figure 4a, when the temperature is 25 °C, the maximum strains in the plastic deformation stage corresponding to the strain rates of 4000 s^−1^,6000 s^−1^, 10,000 s^−1^, and 12,000 s^−1^ are 0.35, 0.42, 0.61, and 0.72, respectively. It can be concluded that under the same compression test device conditions, the plastic deformation stage at room temperature increases significantly with the increase in strain rate. According to Figure 4b–f, under high-temperature deformation conditions (200 °C, 400 °C, 600 °C, 700 °C, 800 °C), compared with the lower strain rate level, the plastic deformation stage of the material under high strain rate conditions also increases significantly. In summary, the PM superalloy has a clear plasticizing effect in the deformation process over a wide range of strain rates.

**Figure 5:**
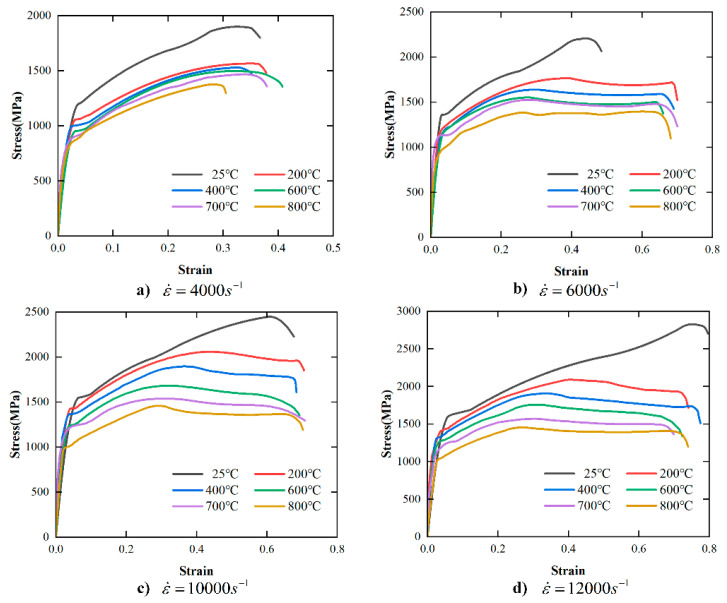
Stress–strain curves of FGH96 superalloy over the wide range of strain rates in the SHPB tests.

The effect of temperature on the stress–strain curve of FGH96 superalloy at different strain rates (4000 s^−1^, 6000 s^−1^, 10,000 s^−1^, 12,000 s^−1^) is shown in Figure 5. According to Figure 5a–d, at the same strain rate, the stress in the plastic deformation stage decreases with the increase in temperature, showing a clear temperature softening effect.

Table 3 shows the yield stress at different temperatures and strain rates under SPHB test conditions. Compared with the yield stress (773 MPa) obtained by the quasi-static compression test at room temperature, the yield stress obtained by SPHB tests at high temperatures and high strain rates increases significantly. When the strain rate is 12,000 s^−1^, the yield strength reaches 1616 MPa. According to Table 3, when the temperature is 200 °C, 400 °C, 600 °C, 700 °C, and 800 °C, the yield strength increases with the increase in strain rate. It is worth noting that the flow stress of the material also increases with the increase in strain rate (Figure 3), which indicates that the PM superalloy material exhibits a significant strain rate hardening effect.

In addition, under the same strain rate loading conditions, the yield strength of the PM superalloy decreases with the increase in temperature. Combined with Figure 4, the flow stress and yield strength decrease with the increase in temperature, which further confirms the temperature softening effect of the PM superalloy.

#### 4.1.2. Strain Hardening

To quantitatively analyze the strain hardening phenomenon of PM superalloy, the strain hardening rate *Q* is calculated by Equation (12).

(12) $$Q_{i}=\frac{\partial \sigma }{\partial \varepsilon }=\frac{\sigma_{i}-\sigma_{i-1}}{\varepsilon_{i}-\varepsilon_{i-1}}$$

where *ε_i_* and represent the strain and the stress of the *i*th experiment, respectively. *ε_i−_*_1_ and *σ_i−_*_1_ represent the strain and the stress of the (*i* − 1)th experiment.

**Figure 6:**
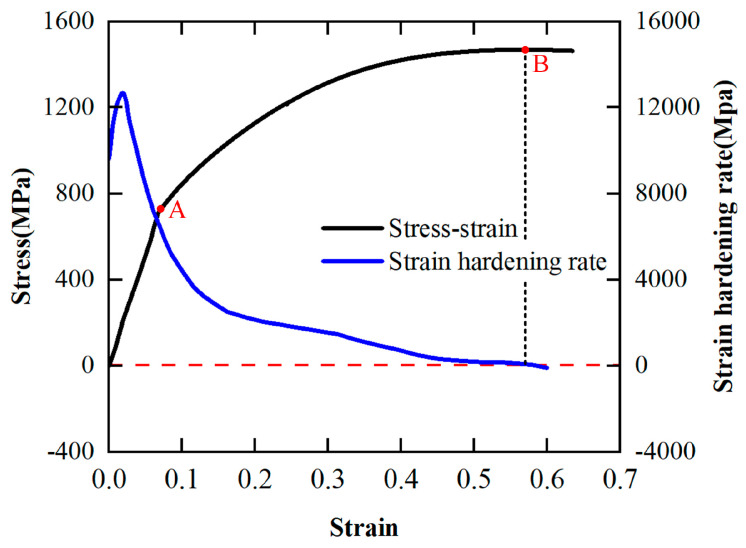
Strain hardening rate–strain curve under quasi-static compression test conditions.

Based on the quasi-static compression experimental data, the strain hardening rate-strain curve of the PM superalloy obtained according to Equation (12) is shown in Figure 6. According to Figure 6, in the elastic deformation stage (*OA*), the strain hardening rate *Q* of the material increases briefly with the increase in strain and then decreases sharply. In the plastic deformation stage (*AB*), the decrease in the strain hardening rate slows down, and at point *B*, it decreases to 0 (it is no longer in the hardening state). In the damage deformation stage, the strain hardening rate is less than 0, and the material is damaged and deformed. Therefore, it can be concluded that during the quasi-static compression test, the strain hardening phenomenon of the FGH96 superalloy is significant, and with the continuous increase in strain, the strain hardening effect gradually weakens and reaches equilibrium at point *B*. This is because in the process of quasi-static compression deformation, with the increase in strain, thermal softening occurs inside the material [22].

**Figure 7:**
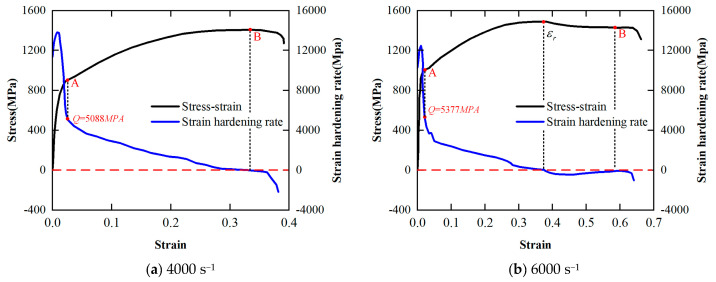
The strain hardening rate–strain curve at 700 °C obtained by SHPB tests.

When the temperature is 700 °C, the strain hardening rate–strain curve of the PM superalloy at different strain rates is shown in Figure 7. According to Figure 7, the PM superalloy still exhibits clear strain hardening under high temperature and high strain rate conditions. However, it is worth noting that in the plastic deformation stage, the strain hardening rate *Q* of the material at the strain rate of 4000 s^−1^ always remains positive. In contrast, the *Q* of the material at the strain rate of 6000 s^−1^ continues to decrease and reaches a negative state after reaching zero. This is because the material not only has the thermal softening effect, but also superimposes the recrystallization softening effect, so that the hardening effect in the plastic deformation stage is greatly weakened, and the flow softening phenomenon appears [23].

#### 4.1.3. Adiabatic Induced Increase in Temperature

According to Section 4.1.1, the PM superalloy has a clear plasticizing effect during high temperature and high strain rate deformation. Researchers have found that the adiabatic temperature rise during material deformation is one of the important factors that cannot be ignored in the plasticizing effect [24]. In addition, the adiabatic temperature rise makes the actual temperature inside the material higher than the experimental loading temperature, which reduces the dislocation slip resistance and strengthens the internal softening of the material. Under the experimental conditions in this paper, the adiabatic temperature rise Δ*T* in the process of material deformation can be obtained by Equation (13).

(13) $$\Delta T=\frac{\eta }{\rho C_{p}}\int_{0}^{\varepsilon }\sigma d\varepsilon$$

where *η* is the plastic work–heat conversion coefficient, for the experimental deformation conditions in this paper, it takes 0.9 [25]; *ρ* is the material density; *C_p_* is the specific heat capacity at atmospheric pressure. The specific heat capacity *C_p_* of the FGH96 superalloy at different temperatures is shown in Table 4.

**Figure 8:**
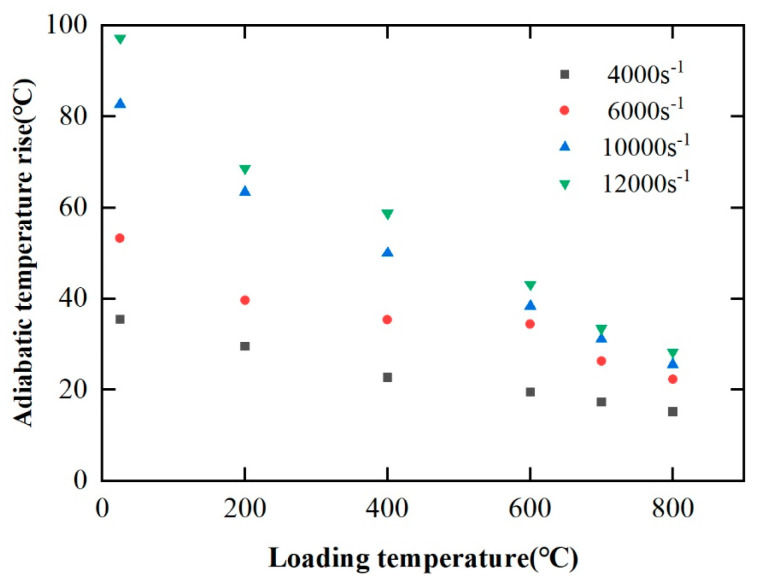
The influence of loading temperature on adiabatic temperature rise during deformation under different strain rates.

**Figure 9:**
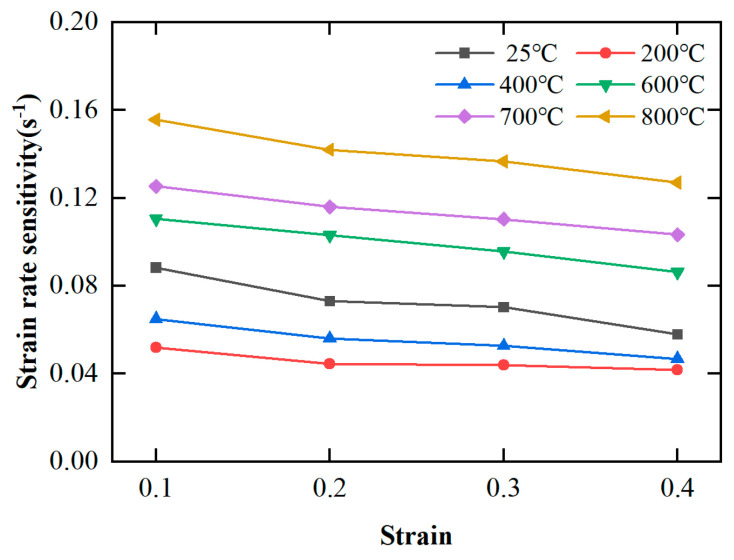
Strain rate sensitivity coefficient–strain curves of FGH96 superalloy at different temperatures.

Figure 8 shows the influence of loading temperature on adiabatic temperature rise of FGH96 superalloy under different strain rates. According to Figure 9, at the same strain rate, the adiabatic temperature rise decreases with the increase in temperature. For example, at the strain rate of 12,000 s^−1^, as the loading temperature increases from 25 °C to 800 °C, the adiabatic temperature rise decreases from 98 °C to 33 °C (reduced by 66%). At the same time, when the strain rate is 4000 s^−1^, the adiabatic temperature rise decreases from 35 °C to 15 °C with the increase in loading temperature (reduced by 60%). It can be seen that with the decrease in strain rate, the rate of adiabatic temperature rise decreases when the increase in loading temperature is reduced. From the above, with the decrease in strain rate, the reduction rate of adiabatic temperature rise decreases significantly with the increase in loading temperature. At the same loading temperature, the higher the strain rate, the higher the adiabatic temperature rise. For example, at 200 °C, the corresponding adiabatic temperature rises at strain rates of 4000 s^−1^, 6000 s^−1^, 10,000 s^−1^, and 12,000 s^−1^ are 30 °C, 40 °C, 65 °C, and 70 °C, respectively. At the same time, compared with the temperatures of 25 °C, 200 °C, and 400 °C, the adiabatic temperature rises at 600 °C, 700 °C, and 800 °C decrease significantly with the increase in strain rate.

#### 4.1.4. The Strain Rate Sensitivity

According to Section 4.1.1, the FGH96 superalloy exhibits a strain rate strengthening effect in the plastic deformation stage. To describe the degree of strain rate strengthening of the material, the strain rate sensitivity coefficient *q* is introduced, as shown in Equation (14). The larger the strain rate sensitivity coefficient *q*, the stronger the strain rate sensitivity of the material.

(14) $$q=\frac{\partial \ln\sigma }{\partial \ln\dot{\varepsilon }}$$

Figure 9 shows the influence of strain on the strain rate sensitivity coefficient of FGH96 superalloy at different temperatures. According to Figure 9, the strain rate sensitivity coefficient decreases with the increase in strain under the experimental temperature conditions. For example, when the temperature is 600 °C, the strain rate sensitivity coefficient decreases from 0.11 to 0.09 with the increase in strain (from 0.1 to 0.4). It is worth noting that the influence of temperature on the strain rate sensitivity coefficient is not clear. For example, for the temperature conditions of 200 °C and 400 °C, the strain rate sensitivity coefficient is lower than the strain rate sensitivity coefficient of 25 °C. However, when the temperature is greater than 600 °C, the strain rate sensitivity coefficient becomes larger and increases with the increase in temperature. At the same time, when the temperature is 800 °C and the strain is 0.1, the largest strain rate sensitivity coefficient is 0.155 (less than 0.2), which shows that FGH96 superalloy exhibits weak strain rate sensitivity.

#### 4.1.5. The Temperature Sensitivity

According to Section 4.1.1, the FGH96 superalloy shows a strong temperature softening effect in the plastic deformation stage. To quantitatively describe the temperature sensitivity of the material, the temperature sensitivity coefficient *s* is introduced, as shown in Equation (15). The greater the temperature sensitivity coefficient *s*, the stronger the temperature sensitivity of the material.

(15) $$s=\left|\frac{\partial \ln\sigma }{\partial \ln T}\right|$$

**Figure 10:**
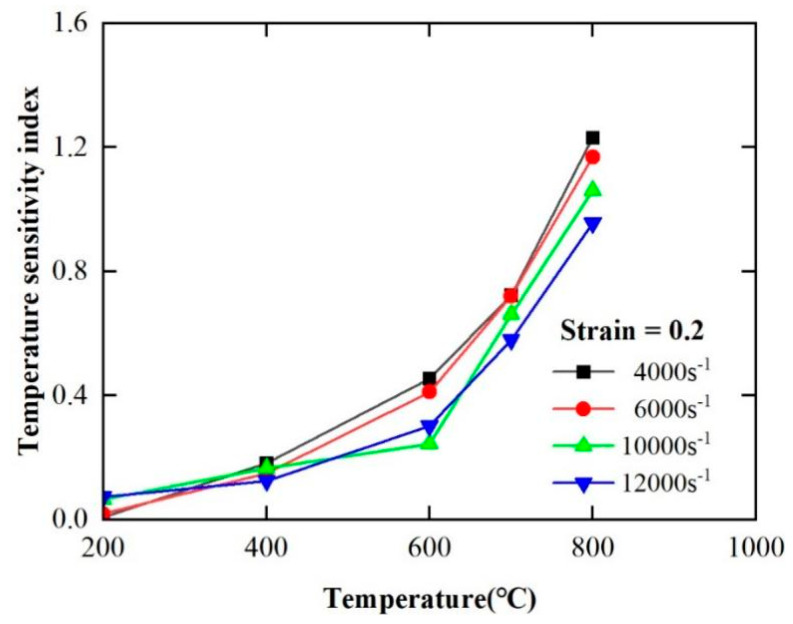
Temperature sensitivity coefficient–temperature curves of FGH96 superalloy at different strain rates.

The variation of the temperature sensitivity coefficient of FGH96 superalloy with temperature at different strain rates calculated by Equation (15) is shown in Figure 10. According to Figure 10, the temperature sensitivity coefficient increases significantly with the increase in temperature under the same strain rate. When the strain rate is 4000 s^−1^, the temperature sensitivity coefficient is 0.1 at 200 °C and reaches 1.2 at 800 °C. At the same time, the temperature sensitivity coefficient decreases with the increase in strain rate. When the temperature is 800 °C, the temperature sensitivity coefficient is 1.3 at 4000 s^−1^ and decreases to 0.9 at 12,000 s^−1^. From the above, the temperature sensitivity of the PM superalloy increases significantly with the increase in temperature and decreases with the decrease in strain rate. In summary, FGH96 superalloy exhibits strong temperature sensitivity.

### 4.2. Construction of Constitutive Model

#### 4.2.1. Recrystallization Critical Condition

**Figure 11:**
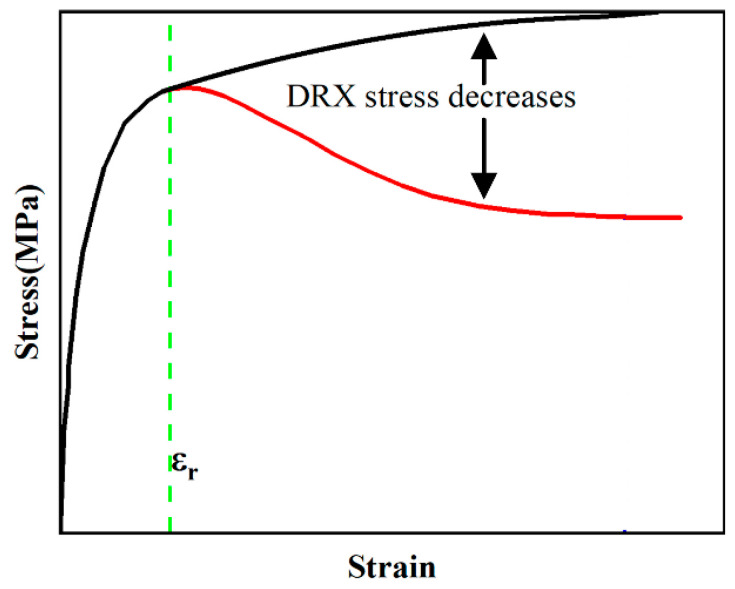
Flow softening of stress–strain curve induced by dynamic recrystallization.

The effect of dynamic recrystallization on the stress–strain curve of the material is shown in Figure 11. If there is no dynamic recrystallization during the deformation process, the flow stress of the material increases slowly with the increase in strain, as shown in the black solid line in Figure 11. When the strain reaches the critical strain of dynamic recrystallization, the flow stress decreases, which is manifested as the recrystallization softening effect as shown in the red line in Figure 10. However, when the strain reaches a certain value (critical strain *ε_r_*), the material undergoes dynamic recrystallization. At this time, the flow stress shows a significant downward trend, as shown in the red solid line in Figure 11, which is the flow softening phenomenon. Therefore, the determination of the critical condition of dynamic recrystallization, namely, the critical strain, is the key to the study of dynamic recrystallization flow softening.

The dynamic recrystallization critical strain of the PM superalloy obtained under the experimental conditions in this paper is shown in Table 5. It can be seen from the data in Table 5 that the critical strain of dynamic recrystallization of FGH96 is not only related to the deformation temperature but also to the strain rate, which verifies the conclusion of Denguir [21]. The critical strain decreases with the increase in temperature and decreases with the increase in strain rate.

**Figure 12:**
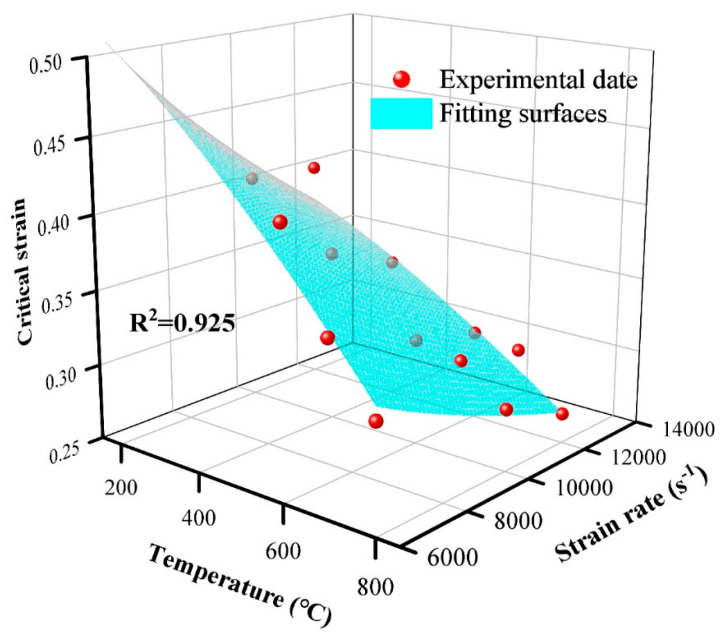
Solving method and results of dynamic recrystallization critical strain.

Based on the data in Table 3, the fitting surface of the critical strain is obtained by polynomial fitting (Equation (11)), as shown in Figure 12.

The equation for calculating the critical strain of dynamic recrystallization of FGH96 under experimental conditions in this paper is shown in Equation (16).

(16) $$\varepsilon_{r}=1.445-5.85\times 10^{-5}T^{1.415}-0.139{\dot{\varepsilon }}^{0.215}+6.45\times 10^{-6}T^{1.415}{\dot{\varepsilon }}^{0.215}$$

#### 4.2.2. Identification of Constitutive Model’s Coefficients

(1) Linear Regression Method

The modified J-C constitutive model (Equation (7)) represents the strain hardening effect, strain rate strengthening effect, thermal softening effect, and recrystallization softening effect from left to right. According to the linear regression parameter solving method, *A*, *B*, *C*, *n*, *m*, and *H_i_* are the parameters to be fitted, *ε*_0_, *T*_0_, and *T_m_* are 0.001 s^−1^, 25 °C, and 1350 °C, respectively.

**Figure 13:**
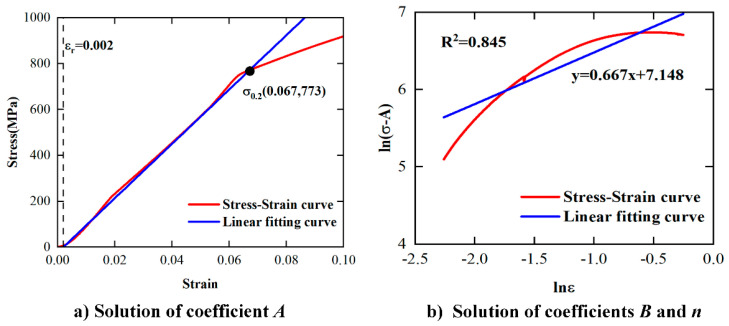
Solution of coefficients *A*, *B*, and *n*.

The strain hardening coefficient can be obtained by processing the quasi-static compression test data at room temperature. The proposed constitutive model is simplified as shown in Equation (17). The quasi-static compression tests permitted to determine the yield stress are represented by coefficient *A* (Figure 13a). Equation (18) was obtained by taking the logarithm on both sides of the Equation (17). The solution of the coefficients *n* and *B* can be obtained by a slope and an intercept of a fitting straight line (Figure 13b). As shown in Figure 12, *A* = *σ*_0.2_ = 773 MPa, *n* = 0.667, *B* = 1271 MPa.

(17) $$\sigma =A+B\varepsilon^{n}$$

(18) ln(σ−A)=nlnε+lnB

The strain rate sensitivity coefficient *C*, the thermal softening index *m*, and the recrystallization softening correction coefficient *H_i_* in the modified J-C constitutive model were obtained from the result of SHPB tests.

**Figure 14:**
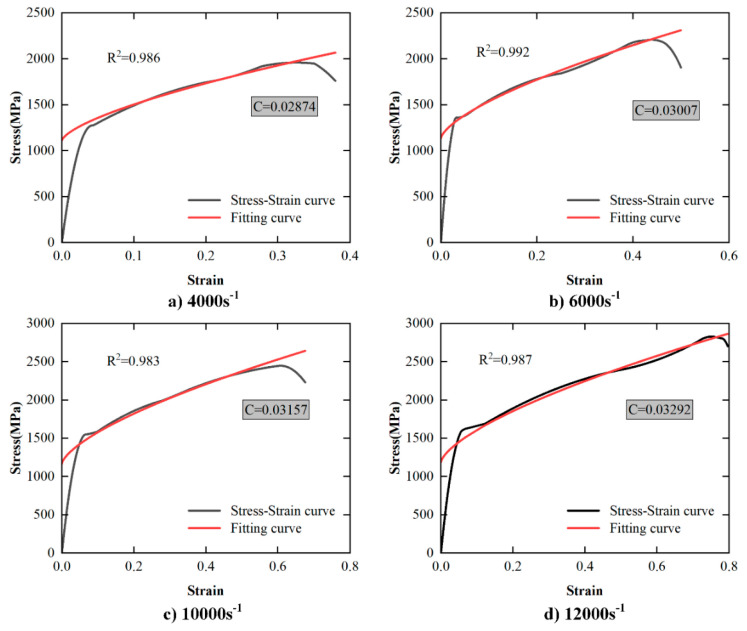
The results of strain rate sensitivity coefficient *C*.

According to SHPB tests at room temperature for different strain rates, the constitutive equation is simplified to Equation (19). The stress–strain curves of materials at different strain rates at room temperature in this paper are shown in Figure 14. The mean value of multiple sets of test results is taken as the final result (*C* = 0.031).

(19) $$\sigma =(A+B\varepsilon^{n})\cdot (1+C\;\ln\frac{\dot{\varepsilon }}{{\dot{\varepsilon }}_{0}})$$

**Figure 15:**
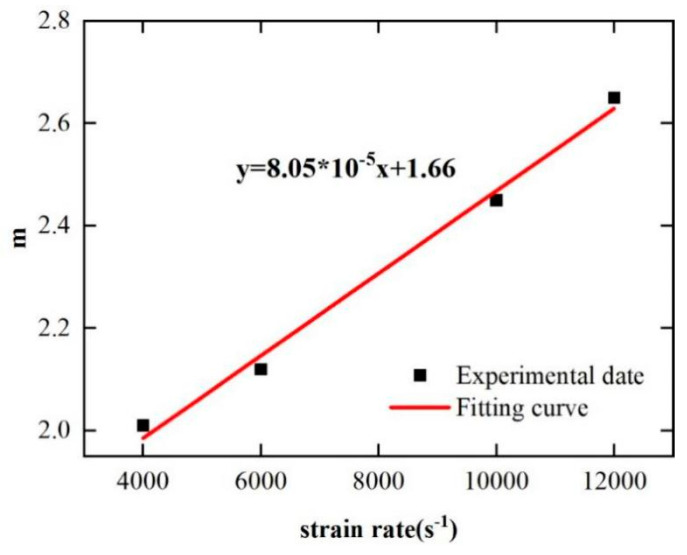
Solution of thermal softening coefficient *m*.

The thermal softening coefficient *m* is determined according to Equation (20). The data of the SHPB tests under high-temperature conditions were used. Finally, the fitting relationship between *m* and strain rate is obtained as shown in Figure 15.

(20) $$m\ln(\frac{T-T_{0}}{T_{m}-T_{0}})=\ln\left(1-\frac{\sigma }{\left(A+B\cdot \varepsilon^{n}\right)\cdot \left(1+C\cdot \ln\frac{\dot{\varepsilon }}{{\dot{\varepsilon }}_{0}}\right)}\right)$$

The coefficients *h_i_* (*i* = 0, 1, 2) related to the dynamic recrystallization can be obtained according to Equation (21). The ratio of the actual flow stress after recrystallization to the predicted stress of the J-C model can be obtained by taking the above parameters into account. The value of parameter *h_i_* (*i* = 0, 1, 2) can be obtained by fitting the experimental results.

(21) $$\frac{\sigma }{f(\varepsilon )f(\dot{\varepsilon })f(T)}=\frac{1}{1-(\frac{h_{0}}{\varepsilon }+h_{1})+(\frac{h_{0}}{\varepsilon }+h_{2})\ln(\frac{\dot{\varepsilon }}{{\dot{\varepsilon }}_{0}})}$$

In summary, the modified constitutive model obtained by the linear regression method is shown in Table 6.

(2) Function iteration method

According to the function iteration method [26,27], the proposed model is shown in Equation (22). The iterative function method determines the coefficients of the prediction model by continuously iterating the function through the optimization algorithm.

(22) $$\sigma =f(\varepsilon )\cdot f(\dot{\varepsilon })\cdot f(T)\cdot f(H_{i})$$

**Figure 16:**
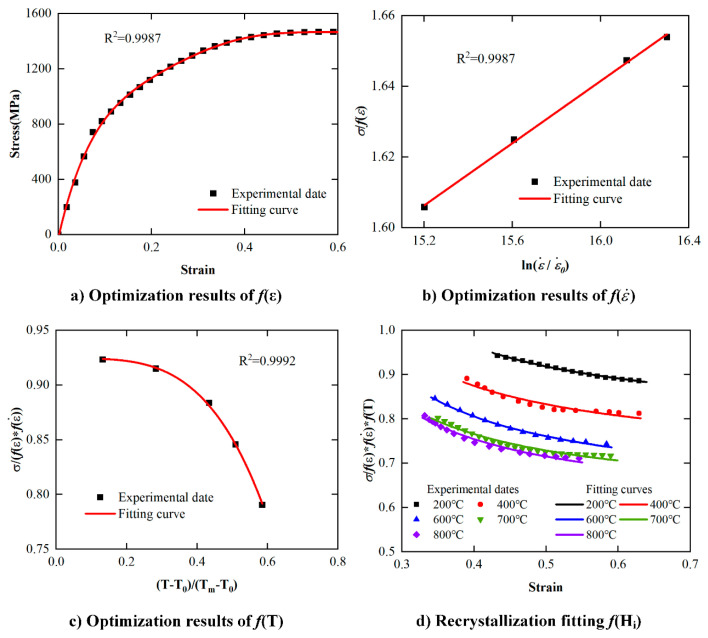
Function iteration method model solution results.

The stress values corresponding to the room temperature *T*_0_ and the reference strain rate *ε*_0_ conditions are selected as the initial values. The quasi-static compression test data at room temperature are selected for polynomial fitting to obtain the results of *f*(*ε*), which are shown in Figure 16a. Then, according to the relationship between measured stress in the SHPB test at room temperature and *f*(*ε*), the relationship between stress and strain rate is obtained by iteration, which is *f*($\dot{\varepsilon }$) as shown in Figure 16b. Finally, the results of *f*(*T*) and *f*(*H_i_*) can be obtained according to the high-temperature SHPB experimental data by the iteration process, as shown in Figure 16c and Figure 16d, respectively.

In the iterative process, the error *R*^2^ is used to judge the accuracy of the results. When the result meets Equation (23), the result is considered to be accepted.

(23) $$\left|R_{k}^{2}-R_{k-1}^{2}\right|\leq 10^{-3}$$

where $R_{k}^{2}$ is the error of determination of the *i*th iterated function,$R_{k-1}^{2}$ is the error of determination of the (*i* − 1)th iterated function.

Finally, the constitutive model constructed by the function iteration method is obtained. The constitutive model after reaching the critical strain of recrystallization is shown in Equation (24).

(24) $$\begin{array}{l} \sigma =(427.05+4856.82\varepsilon -7502.10\varepsilon^{2}+4053.41\varepsilon^{3}-381.92\varepsilon^{4})\\ \times (0.969+0.042ln(\frac{\dot{\varepsilon }}{{\dot{\varepsilon }}_{0}}))\times (0.924-{\left(\frac{T-T_{0}}{T_{m}-T_{0}}\right)}^{3.768})\\ \times {\left[\left(1-(-0.008/\varepsilon +7.094)+(-0.008/\varepsilon +0.479)\ln(\frac{\dot{\varepsilon }}{{\dot{\varepsilon }}_{0}}\right)\right]}^{-1} \end{array}$$

(3) Comparison of different methods

**Figure 17:**
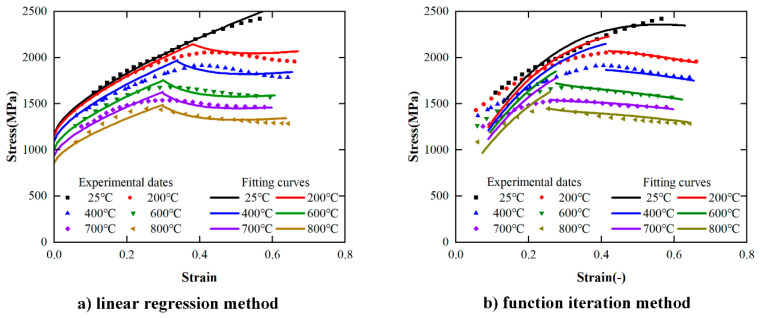
Comparison of experimental results and predicted results ($\dot{\varepsilon }=10,000s^{-1}$).

To optimize the solution method of the parameters, the stress–strain curves obtained by the constitutive equations solved by the two methods described in the previous section are compared with the experimental results. Under the condition of strain rate is 10,000 s^−1^ and the temperature is 25 °C, 200 °C, 400 °C, 600 °C, 700 °C, 800 °C, the comparison results of stress–strain curves are shown in Figure 17. Figure 17a,b are the experimental comparison results of the linear regression solving method and functional iteration solving method, respectively. According to Figure 16, both of the equations obtained by the linear regression solving method and functional iteration method can describe the trend of stress in the plastic deformation stage.

**Figure 18:**
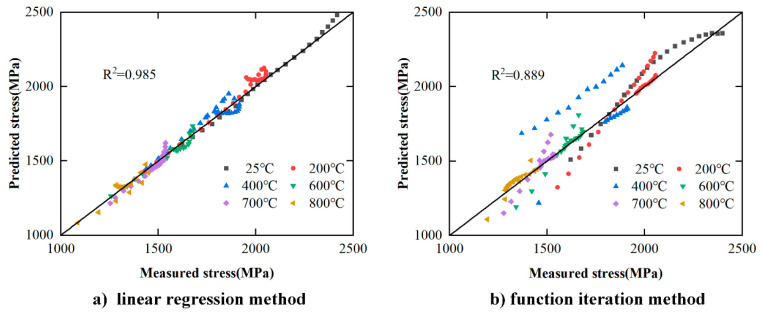
Comparison of correlation between predicted stress and measured stress by different solving methods.

To quantitatively evaluate the overall error of the two methods, the scatter plot is used to calculate the correlation value. The results of the correlation between the calculated stress and the experimental stress obtained by the two methods are shown in Figure 18. Figure 18a,b are the results of the linear regression solving method and functional iteration solving method, respectively. Compared with Figure 18b (*R*^2^ = 0.889), the data correlation index in Figure 18a is higher (*R*^2^ = 0.985), namely, the data concentration is higher.

The maximum relative error *θ* between the measured and predicted stress is also calculated to evaluate prediction accuracy, as shown in Equation (25).

(25) $$\theta =\max\left(\frac{\left|\sigma_{p}-\sigma_{m}\right|}{\sigma_{m}}\times 100\%\right)$$

where *σ_p_* is the predicted stress, *σ_m_* is the measured stress.

The maximum relative error of the constitutive model obtained by the linear fitting method and the function iteration method are shown in Table 7. According to Table 7, the accuracy of the model obtained by the linear regression method (11.21%) is much higher than that obtained by the function iteration method (4.74%) in the plastic deformation of the non-dynamic recrystallization stage. Correspondingly, in the recrystallization stage, the accuracy of the model obtained by the function iteration method is improved (4.11%), which is very small compared with the accuracy of the model obtained by the linear regression method (5.11%). By calculating the average value of the maximum error $\bar{\theta }$ before and after dynamic recrystallization, the model accuracy obtained by the linear regression method is greater than the model obtained by the functional regression method in the whole plastic deformation stage.

In summary, the modified J-C constitutive equation considering the recrystallization softening effect proposed in this paper is solved by the linear regression method, and the results are shown in Equation (26).

(26) $$\begin{array}{l} \sigma =(773+1271\varepsilon^{0.667})\times (1+0.031ln(\frac{\dot{\varepsilon }}{{\dot{\varepsilon }}_{0}}))\times \left(1-{\left(\frac{T-T_{0}}{T_{m}-T_{0}}\right)}^{1.66+8.05\times 10^{-5}\dot{\varepsilon }}\right)\\ \times {\left[\left(1-(-0.015/\varepsilon -0.015)+(-0.015/\varepsilon +0.046)\ln(\frac{\dot{\varepsilon }}{{\dot{\varepsilon }}_{0}}\right)\right]}^{-1} \end{array}$$

### 4.3. Validation of Modified J-C Constitutive Model

Temperature-dependent J-C model [28,29] is also widely used to predict the flow stress behavior of materials in the cutting process, which is shown in Equation (27).

(27) $$\sigma =\left(A+B\varepsilon^{n}\right)(1+C\ln\frac{\dot{\varepsilon }}{{\dot{\varepsilon }}_{0}})\left[1-{\left(\frac{T-T_{0}}{T_{m}-T_{0}}\right)}^{m}\right]\left(\frac{1}{1-(a-b\varepsilon )}\right)$$

where *a* and *b* are the coefficients related to flow softening caused by dynamic recrystallization. According to the stress–strain data of the plastic deformation stage of the FGH96 under the experimental conditions in this paper, the temperature-dependent J-C model for the plastic deformation stage after reaching the recrystallization critical strain is shown in Equation (28).

(28) $$\begin{array}{l} \sigma =(773+1271\varepsilon^{0.667})(1+0.031\ln\frac{\dot{\varepsilon }}{{\dot{\varepsilon }}_{0}})\left(1-{\left(\frac{T-T_{0}}{T_{m}-T_{0}}\right)}^{1.66+8.05\times 10^{-5}\dot{\varepsilon }}\right)\left(\frac{1}{1-(0.274-1.234\varepsilon )}\right) \end{array}$$

**Figure 19:**
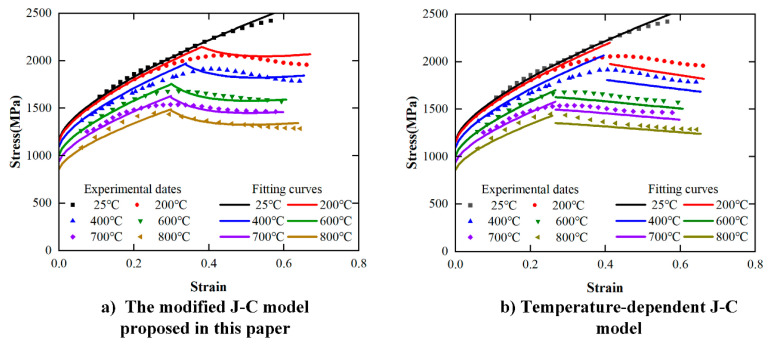
Comparison of correlation between predicted stress and measured stress by two models.

The comparison between the predicted stress and the experimental stress of the two modified models at the 10,000 s^−1^ strain rate is shown in Figure 19. Figure 19a,b are the comparison results of the model proposed in this paper and the temperature-dependent J-C model, respectively. The temperature-dependent J-C model can also predict the flow stress in the plastic deformation stage.

**Figure 20:**
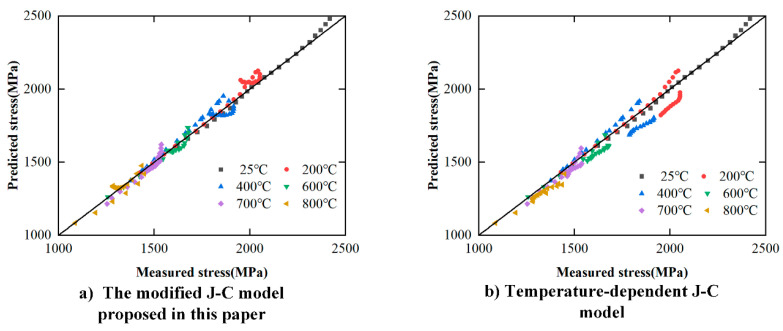
Comparison of correlation between predicted stress and measured stress by different modified models.

The results of the correlation analysis between the calculated stress and the experimental stress obtained by the two models are shown in Figure 20. Figure 20a,b are the correlation analysis results of the model proposed in this paper and the temperature-dependent J-C model, respectively. Compared with Figure 20b (*R*^2^ = 0.956), the data correlation index in Figure 20a is higher (*R*^2^ = 0.985), namely, the data concentration is higher.

According to Equation (25), the average values of the maximum relative error of the two models were calculated. The average value of the maximum relative errors of flow stress predicted by the modified J-C model in this paper and the temperature-dependent J-C model is 5.11% and 8.14%, respectively. Therefore, the constitutive model proposed in this paper can accurately predict the flow stress of FGH96 in the plastic deformation stage under the influence of recrystallization softening.

## 5. Conclusions

In this work, the dynamic mechanical properties of nickel-based PM superalloy FGH96 are analyzed by quasi-static compression tests and SHPB tests at strain rates of 4000 s^−1^~12,000 s^−1^ and temperatures of 25 °C~800 °C. A J-C constitutive model considering the dynamic recrystallization softening effect is proposed. The following conclusions can be drawn in this paper.

(1) Dynamic mechanical properties of nickel-based PM superalloy were obtained by experiments. The PM superalloy has a clear plasticizing effect in the deformation process over a wide range of strain rates. The PM superalloy material exhibits a significant strain rate hardening effect and temperature softening effect. During the compression test, the strain hardening phenomenon of PM superalloy is significant and the strain hardening effect gradually weakens with the continuous increase in strain. FGH96 superalloy exhibits weak strain rate sensitivity and strong temperature sensitivity.
(2) Considering the influence of temperature and strain rate, the formula for the flow stress softening term $H(\varepsilon ,\dot{\varepsilon },T)$ and critical strain *ε_r_* of dynamic recrystallization were obtained. On this basis, the modified J-C constitutive model of PM superalloy considering dynamic recrystallization behavior in a wide range of strain rates and temperature is proposed.
(3) The coefficients of the modified constitutive equation established in this paper were obtained by linear fitting and function iteration, respectively. Through correlation analysis and maximum error analysis, the modified constitutive model solved by the linear fitting method has higher accuracy in predicting flow stress in the plastic deformation stage.
(4) Compared with the temperature-dependent constitutive model, it is found that the modified constitutive model established in this paper is more suitable to describe the recrystallization softening linearity of flow stress at high temperature and high strain rate in the plastic deformation stage of powder metallurgy superalloy.

## Figures and Tables

**Table 1:** Chemical composition of FGH96 superalloy.

Element	C	Al	Ti	Cr	Co	Nb	Mo	W	Ni
Wt. (%)	0.03	2.2	3.7	16	13	0.8	4	4	Bal

**Table 2:** Experimental conditions of SHPB tests.

Variable	Values
Strain rate $\dot{\varepsilon }$ (s^−1^)	4000, 6000, 10,000, 12,000
Temperature *T* (°C)	25, 200, 400, 600, 700, 800

**Table 3:** Yield strength at different temperatures and strain rates (MPa).

Strain Rate (s^−1^)	Temperature, *T* (°C)					
	25	200	400	600	700	800
**4000**	1315	1305	1234	1097	903	883
**6000**	1359	1318	1280	1142	1001	926
**10,000**	1544	1487	1322	1227	1182	994
**12,000**	1616	1588	1355	1271	1205	1043

**Table 4:** Specific heat capacity of FGH96 superalloy at different temperatures.

Temperature, *T* (°C)	25	200	400	600	700	800
*C_p_* (kJ/ (kg‧°C))	0.391	0.422	0.455	0.487	0.503	0.525

**Table 5:** Critical strain *ε_r_* under different temperature and strain rate conditions.

Strain Rate (s^−1^)	Temperature, *T* (°C)				
	200	400	600	700	800
6000			0.4233	0.3621	0.3211
10,000	0.4033	0.3640	0.3199	0.3144	0.2911
12,000	0.3999	0.3443	0.3091	0.3051	0.2698

**Table 6:** Constitutive model coefficients of linear regression method.

Coefficients	Value
*A* (MPa)	773
*B* (MPa)	1271
*C*	0.031
*n*	0.667
*m*	$8.05\times 10^{-5}\dot{\varepsilon }+1.66$
*h*_0_	−0.015
*h*_1_	−0.015
*h*_2_	0.046
$\varepsilon_{r}$	$1.445-5.85\times 10^{-5}T^{1.415}-0.139{\dot{\varepsilon }}^{0.215}+6.45\times 10^{-6}T^{1.415}{\dot{\varepsilon }}^{0.215}$

**Table 7:** Comparison of the maximum relative errors of two different methods.

Maximum Relative Errors	Linear Regression Method	Function Iteration Method
$\theta_{1}(\varepsilon <\varepsilon_{r})$	4.74%	11.21%
$\theta_{2}(\varepsilon \geq \varepsilon_{r})$	5.11%	4.11%
$\bar{\theta }$	4.93%	7.66%

## Data Availability

Data are contained within the article.
